# Care demands and professional commitments of parents of children with specific learning disabilities: a systematic review

**DOI:** 10.3389/fpubh.2026.1705478

**Published:** 2026-02-27

**Authors:** Telang Monika Vitthal, Akriti Srivastava, Ravinder Kumar, Rameshbabu Tamarana, Sonali Mukherjee

**Affiliations:** 1 Department of Psychology, Central University of Karnataka, Kalaburagi, India; 2 Central University of Andhra Pradesh, Anantapur, India; 3 Central University of Karnataka, Kalaburagi, India; 4 CHRIST (Deemed to be University), Bengaluru, India

**Keywords:** parental demands, parenting challenges, professional commitments, specific learning disabilities, work-life balance

## Abstract

**Background:**

Balancing employment responsibilities and care demands of parents of children with specific learning disabilities (SLDs) often creates a challenging situation. This systematic review synthesizes and evaluates studies to elucidate the complex challenges these parents face in balancing their professional work and parental duties.

**Objective:**

This study aimed to synthesize the existing literature on the challenges faced by parents to effectively manage both their professional commitments and the care demands of their children with specific learning disabilities.

**Methods:**

A variety of databases, including PubMed, Web of Science, and ScienceDirect, were used. A manual search was also conducted using Google Scholar and ResearchGate to identify additional studies. This review focused on studies conducted between January 1995 and May 2025, involving working parents (mothers and fathers) of children with specific learning disabilities under the age of 18. A total of 15 studies met the eligibility criteria for inclusion. The methodological quality was assessed using the Critical Assessment Checklist developed by the Joanna Briggs Institute.

**Results:**

The results show that parents experience various challenges, including elevated stress levels and difficulty in connecting with support networks, while navigating societal perspectives on their child’s condition. A wide range of coping skills was identified, such as using emotion-focused strategies, seeking social support, and employing various parenting approaches. Thus, the way parents balance their roles as both caregivers and employees is influenced by societal expectations and cultural norms.

**Conclusion:**

The literature emphasizes the challenges parents face in balancing their dual roles as employees and carers. The analysis highlights the need to develop parental-mediated interventions, legislation, and supportive environments to help parents effectively manage their dual roles.

## Introduction

Over a billion individuals, or approximately 15% of the global population, have some form of disability (1). Within this demography, specific learning disabilities (SLD) is a prominent category, affecting approximately 10% of children worldwide (1). According to the American Psychiatric Association (2), SLD frequently occurs alongside other challenges, affecting 5–15% of school-age children globally.

It is essential to distinguish SLD from generalized intellectual disabilities (ID). Unlike ID, SLD is characterized by persistent difficulties in specific academic areas, such as reading or mathematics, in individuals who typically have average to above-average intellectual functioning (2). As a result, this review focuses primarily on the challenges encountered by parents of children with SLD.

Compared to parents of typically developing children, parents of children with SLD often experience greater physical and emotional distress. Most often, they internalize feelings of guilt for their child’s condition, which lead to anxiety and depression regarding the child’s long-term prospects (3). According to recent findings, after receiving a child’s diagnosis, parents often get triggered by a phase of initial shock, which leads to emotional dysregulation (4). This disconnect between parental expectations and the reality of the situation can lead to feelings of grief, ultimately affecting the family’s dynamics and overall quality of life (5).

Parental responsibility also impacts work engagement, particularly for women (6). This framework is understood through two fundamental concepts: work–family conflict (WFC), where work responsibilities interfere with family life, and family–work Conflict (FWC), where parenting duties interfere with work productivity. This issue has become more noticeable as more women enter the workforce (7). Despite increased attention to gender equality in today’s contemporary society, the majority of child care responsibilities is still provided by women, creating substantial obstacles in managing caregiving duties and professional commitments (8). This creates significant stress, particularly among working mothers compared to non-working mothers (9). Stress levels are influenced by factors such as educational attainment and family size (10). In addition, a recent economic analysis shows that parental labor market engagement is negatively correlated with the severity of a child’s impairment (11).

Al-Yagon and Cinamon (12) found that mothers of children with SLD had lower levels of work–family conflict (WFC) but higher levels of family–work conflict (FWC) than mothers of children without SLD. This is likely because SLD is “invisible” and requires parents to participate in rigorous academic remediation and to advocate on their child’s behalf. Taking on dual responsibilities often results in increased work demands, low self-esteem, depression, and a lack of motivation (13).

In particular, the care demands of children with SLD, such as regular school meetings and homework supervision, consume substantial time and cognitive capacity that could otherwise be devoted to professional development (12, 14). This depletion of resources results in inter-role conflict, where the demands of one role make it difficult to fulfill the commitments of the other, according to conflict theory (14). According to the current empirical study, this dynamic directly affects professional commitment by compelling structural shifts in employment. For instance, Wondemu et al. (11) stated a direct correlation between lower parental income and professional obligations and parenting duties related to disabilities that restrict career growth opportunities.

Although developmental disabilities such as autism and cerebral palsy have been extensively explored, there is a paucity of research regarding the challenges faced by parents of children with learning disabilities (LD) in balancing professional commitments and parental demands. This underscores the relevance of this study. Given the limited number of studies focusing on the LD population, foundational works and broader disability literature have been incorporated to provide unique insights into the dynamics of employment and caregiving. Additionally, this review focuses on parents of children under the age of 18 because the caregiving requirements for adult children differ significantly from those for minors (15).

Given the above-mentioned facts, it is essential to examine the challenges parents face in balancing professional commitments with caregiving demands for children with LD. This analysis provides vital information for the development of parental-mediated interventions, supportive policies, and targeted services.

## Objective

This study aimed to synthesize the existing literature on challenges faced by parents to effectively manage both their professional commitments and the care demands of their children with SLD. Beyond analysis, this review outlines implications for workplace policy and clinical practice and identifies directions for further investigation to close the systemic gaps in the currently available information.

## Methods

### Eligibility criteria

This review includes studies that employ mixed, qualitative, and quantitative methods. It includes research published in English between January 1995 and May 2025. The participants in these study were parents (both fathers and mothers) of children diagnosed with SLD who are under the age of 18. The study participants were required to hold a paid part-time or full-time job. The research examined how work–life balance, employment status, and professional obligations are related to caregiving demands.

Studies were excluded if parents of children with SLD were not employed and if the children were older than 18 years. Previous studies have indicated that providing care for adult children with disabilities differs from caring for minors (15). Non-peer-reviewed sources, including dissertations, conference proceedings, and gray literature, were excluded from the review. Additionally, studies were excluded if they investigated other developmental disabilities (e.g., autism spectrum disorder and ID) without providing detailed information specific to the SLD group; however, studies could be included if comprehensive data specific to the SLD subgroup were available for analysis.

### Information sources

A systematic search of the following databases was conducted: ScienceDirect, Web of Science, and PubMed. Additional manual searches were conducted using Google Scholar and ResearchGate to identify studies that might have been overlooked or not indexed in the central databases in order to ensure comprehensive coverage. In addition, published research was identified by searching the reference lists of relevant studies and review articles.

### Search strategy

To maintain consistency across databases (e.g., PubMed and ScienceDirect), every search included free-text keywords rather than specific database subject headings [e.g., Medical Subject Headings (MeSH)]. To enable the retrieval of studies published through May 2025, all electronic database searches were conducted simultaneously. The search strategy includes the following terms based on a Boolean structure: (“Specific Learning Disability” OR “Dyslexia” OR “Learning Disability” OR “Learning Disorder”) AND (“Parents” OR “Mothers” OR “Fathers” OR “Caregivers”), AND (“Employment” OR “Work–Family Balance” OR “Work–Life Balance” OR “Professional Commitment” OR “Career”).

### Study selection and data extraction

To identify eligible articles, two reviewers independently screened the studies using inclusion and exclusion criteria. Zotero was used to save the studies, export them to MS Excel for screening, and remove any duplicate studies. The reviewers conducted both primary and secondary screenings of all the extracted studies for the systematic review. Reasons for excluding studies were noted, and data were extracted from the selected studies. To maintain conceptual consistency, data extraction for studies using mixed samples of developmental disorders was specifically restricted to disaggregated qualitative or quantitative findings related to the specific subgroup of SLD. The reference lists of the selected studies were manually scanned to ensure that no articles were missed for inclusion. Disagreement over which study to include was resolved through discussion and consensus with a third party.

### Risk of bias assessment

To reduce the potential risk bias, eligible studies were critically assessed by reviewers at the study level using the Joanna Briggs Institute (JBI) Critical Appraisal Checklist Cross-Sectional Design (16), JBI Critical Appraisal Checklist for Cohort Studies (37), and JBI Critical Appraisal Checklist for Qualitative Research (17). All items have three potential responses: “yes,” “unclear,” and “no,” with “yes” scoring 1, and the others 0. Once again, any disagreements between the reviewers were resolved through discussion. Following the critical appraisal, a threshold was established, and studies were classified as “low risk” only if they met more than 70% of the checklist requirements (18). This decision was based on the reviewers’ overall assessment of quality and risk of bias. While studies met the predefined threshold, inherent design limitations remain and should be considered when interpreting the findings.

### Synthesis of results

The eligibility of the studies for the systematic review was determined using the Preferred Reporting Items for Systematic reviews and Meta-Analyses (PRISMA) 2020 flow diagram. Significant methodological heterogeneity, including studies with diverse research designs, geographic contexts, and outcome measures, prevents a statistical meta-analysis. Therefore, a narrative synthesis was conducted to identify the difficulties parents encounter in balancing their professional commitments with the care demands of their children with SLD, following the guidance of Popay et al. (19). Data extraction employed an inductive coding approach to systematically identify convergent patterns, yielding five distinct themes.

## Results

### Study selection

The initial search yielded 5,397 articles from primary-indexed databases using the specified keywords, of which 1,424 were from ScienceDirect, 2,973 from PubMed, and 1,000 from Web of Science. Supplemental searches yielded 336 articles, among which were 40 studies from ResearchGate and 296 from Google Scholar. This led to the identification of 5,733 articles in total. After removing 323 duplicates, 5,410 articles remained for screening. Following screening for inclusion and exclusion criteria, 32 publications were selected for in-depth full-text analysis after 5,378 records were excluded.

Of the 32 studies identified, 24 were successfully retrieved and evaluated for eligibility. Following the database search, 13 articles were excluded for the following reasons: intervention studies (*n* = 3), not relevant to the population (*n* = 5), not specific to the topic (*n* = 4), and review papers (*n* = 1), leaving 11 studies included.

**Figure 1:**
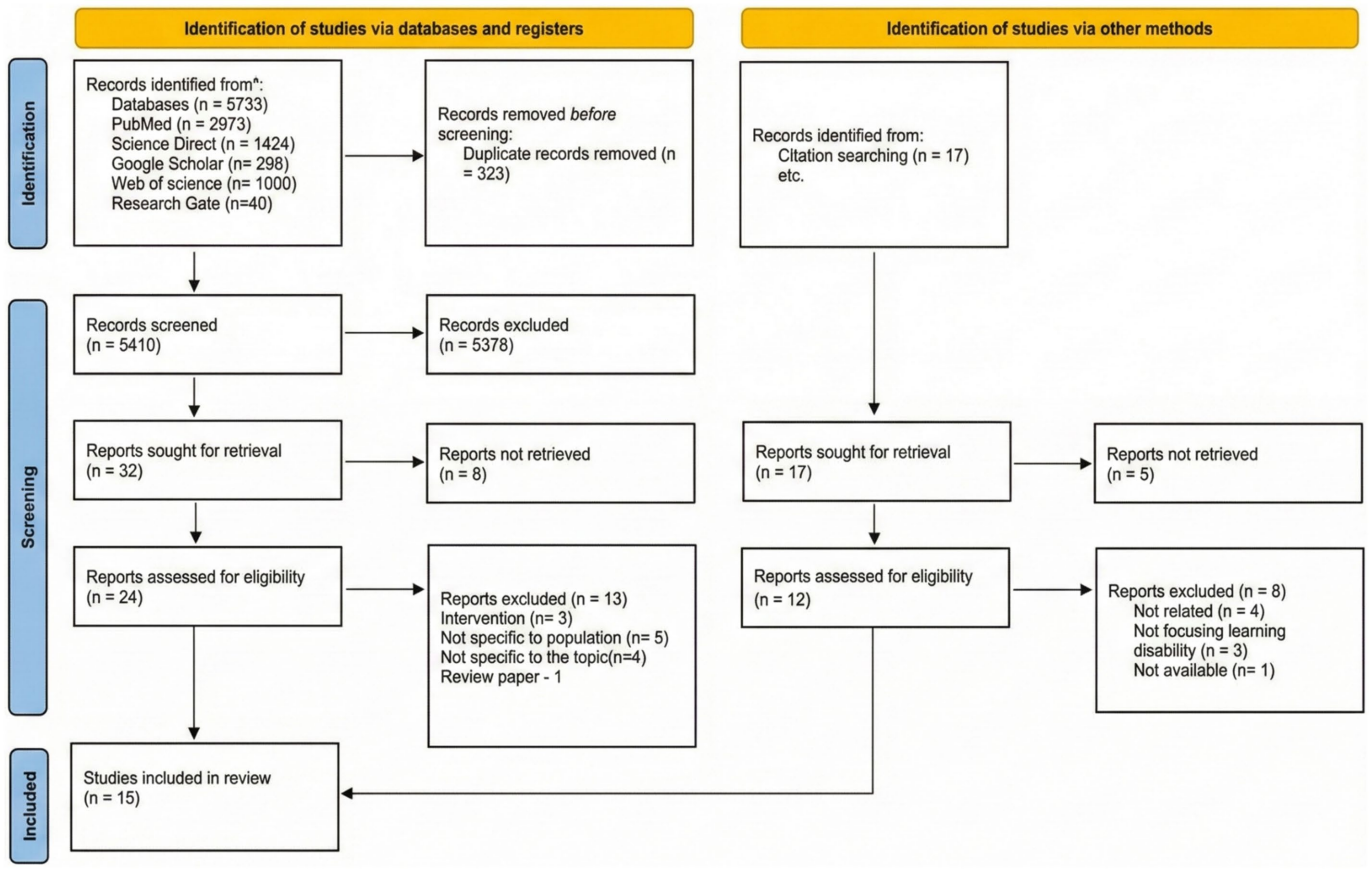
PRISMA flowchart detailing the study selection process.

Additionally, manual searches were conducted to identify 17 records; 12 of these records were retrieved for further analysis. Among these, 4 records met the inclusion criteria, and 8 were excluded for reasons such as, not being related to the working parents (*n* = 4), not focusing on SLD (*n* = 3), and full text being inclusion criteria, and 8 were excluded for the following reasons: not related to working parents (*n* = 4), not focusing on SLD (*n* = 3), and full text unavailable (*n* = 1). In the final analysis, this systematic review included 15 studies (Figure 1). Authors’ names, publication year, sample size, study design, and findings were among the details extracted.

### Study characteristics

The studies conducted shed light on the difficulties parents face in balancing the care demands of their children with SLD with their professional responsibilities. There were 1,580 participants in all 15 studies, including one that only included mothers of children with SLD (*n* = 96) (12). The studies were carried out in the United States (*n* = 4), Malaysia (*n* = 2), South Africa (*n* = 1), India (*n* = 4), Taiwan (*n* = 1), Canada (*n* = 1), and Israel (*n* = 2).

Regarding study methodology, most studies employed a cross-sectional design (20–23). Other designs included longitudinal (24), phenomenological (25), and exploratory (26). All included studies were published between 1995 and 2022. The characteristics of all 15 studies are detailed in Table 1.

**Table 1:** Study characteristics of care demands and professional commitments of parents of SLD children.

Author(s)/Year/Location	Objectives	Design/Sample	Key findings
1. Freedman et al. (26) USA	To acquire information on how caregiving affects their roles at home and work from parents of children with developmental disabilities.	Exploratory (Qualitative) N: 26 (Data specific to SLD parents extracted)	Parents who work gain benefits emotionally, financially, and in terms of autonomy and purpose.
2. Anuar et al. (20) Malaysia	To assess the coping mechanisms and stress levels of parents of children with SLD in light of their characteristics.	Cross-sectional N: 274	No gender difference in the stress levels. Stress levels vary by socioeconomic status (SES), educational background, and race. Parents often adopt emotion-based coping strategies.
3. Taderera and Hall (25) Namibia	To look into the challenges encountered by parents of SLD children in Opuwo, Namibia.	Phenomenological N: 8	Lack of knowledge and unstable finances are the main challenges, especially for single, unemployed parents.
4. Sreedevi (30) India	To assess the needs of parents of SLD children.	*Ex post facto* N: 60	A significant challenge is financial instability. Lack knowledge of the resources.
5. Paul and Babu (28) India	To assess how parents and parenting practices affect the development of life skills in children with SLD.	Cross-sectional N: 100	Parenting styles (responsive, domineering, and neglectful) and family income affect development. Responsive parenting has a positive impact on children’s life skills.
6. Mitchell (24) UK	To investigate the factors that parents of children with SLD take into account when making important choices about their own and their children’s roles in contexts such as social care, education, and health.	Longitudinal (Qualitative) N: 14	Parental decision-making is influenced by personal beliefs, safety concerns, and trust in professionals.
7. Simon and Easvaradoss (38) India	To investigate parenting stress and quality of life among parents of children with SLD.	*Ex post facto* N: 100	Higher stress levels negatively impact the parental quality of life and the child’s academic performance, social relationships, and emotional adjustment.
8. Chang and Hsu (27) Taiwan	To explore how Taiwanese parents’ views about raising children with SLD and their own culture affect parents’ spiritual experiences.	Cross-sectional N: 117	SLD children exacerbate family stress. Parents internalize negative emotions. Informal social support (friends/ family) is valued over the formal support system (professional help).
9. Auriemma et al. (21) USA	To examine the association between parental beliefs, stress, and coping strategies in parents of children with SLD.	Cross-sectional N: 147	Parents endure significantly higher levels of stress that are correlated with the severity of the disability and their use of emotion-focused coping strategies.
10. Dyson (22) Canada	To investigate the unexpected effects that children with learning difficulties have on their families’ lives.	Cross-sectional N: 11	Families face unanticipated consequences, including parental guilt, marital problems, and increased stress.
11. Josephine et al. (36) India	To assess the mean levels of parental distress and difficult child behaviors among parents of children with SLD.	Cross-sectional N: 80 (SLD subgroup analyzed)	Parents of children with neurological disorders experience more stress than parents of typically developing children.
12. Al-Yagon and Cinamon (12) Israel	To examine the impact of raising a child with SLD on the family unit, with particular focus on working mothers’ involvement in the work–family dynamic.	Cross-sectional N: 96	Mothers experience a higher level of FWC but lower levels of WFC.
13. Kamaruddin et al. (29) Malaysia	To evaluate the degree of stress encountered by the parents of children with SLD.	Cross-sectional N: 264	Mothers report more stress than fathers. Stress levels correlate with higher SES.
14. Towers (31) UK	To study the experiences of fathers raising disabled children to inform practices and policies that value the father’s contribution to families raising SLD children.	Cross-sectional N:251	60% of fathers spend more time with their child. The majority of them were unaware of unpaid leave.
15. Heiman (23) Israel	To study parents’ perspectives of children with learning, physical, or intellectual disabilities based on family resilience traits.	Cross-sectional N: 32 (SLD subgroup analyzed)	The majority of the parents experienced social life modifications. Coping mechanisms involve hope, acceptance, and trust.

### Risk of bias in studies

Table 2.

**Table 2:** Risk of bias assessment for studies (*n* = 15).

S. No.	Studies	Study design (JBI tool used)	Total scores	Risk of bias
1	Freedman et al. (26)	Qualitative	8/10 (80%)	Low
2	Anuar et al. (20)	Cross-sectional	6/8 (75%)	Low
3	Taderera and Hall (25)	Qualitative	8/10 (80%)	Low
4	Sreedevi (30)	*Ex post facto* (Cross-sectional)	6/8 (75%)	Low
5	Paul and Babu (28)	Cross-sectional	6/8 (75%)	Low
6	Mitchell (24)	Qualitative	8/10 (80%)	Low
7	Simon and Easvaradoss (38)	*Ex post facto* (Cross-sectional)	6/8 (75%)	Low
8	Chang and Hsu (27)	Cross-sectional	6/8 (75%)	Low
9	Auriemma et al. (21)	Cross-sectional	6/8 (75%)	Low
10	Dyson (22)	Cross-sectional	6/8 (75%)	Low
11	Josephine et al. (36)	Cross-sectional	6/8 (75%)	Low
12	Al-Yagon and Cinamon (12)	Cross-sectional	6/8 (75%)	Low
13	Kamaruddin et al. (29)	Cross-sectional	6/8 (75%)	Low
14	Towers (31)	Cross-sectional	6/8 (75%)	Low
15	Heiman (23)	Cross-sectional	6/8 (75%)	Low

#### Result synthesis

1. Parenting difficulties and Stressors: Families who have children with SLD deal with unanticipated consequences, both good and bad, such as parental guilt, marital problems, and elevated stress levels. The presence of children with SLD causes unanticipated changes in family relations, which give rise to coping mechanisms (22). However, another study found that these difficulties had a detrimental effect on a child’s ability to adjust emotionally, engage socially, and function academically. Parents of children with SLD reported feeling more stressed out and having a poorer quality of life (38).
2. Coping strategies and Resilience: Parents of children with SLD encounter more stress, irrespective of gender, with families from higher socioeconomic and educational backgrounds relying on emotion-based coping (20). There are also racial, occupational, and educational differences in stress levels. Loneliness and isolation are often caused by social exclusion and the loss of social connections (27). Resilience remains evident regardless of these difficulties. Heiman (23) indicated that, while parents experienced changes in their social lives, frustration and dissatisfaction underscored the importance of maintaining hope, acceptance, and trust in their child and their future. Comparable findings noted that parenting stress levels are linked with the severity of the child’s SLD and the application of emotion-focused coping techniques (21).
3. Parenting Styles and Developmental Milestones: Parenting approaches have a significant impact on child development, regardless of the fact that they often lack resources. Hence, family income substantially affects the development of essential life skills among children with SLD (28). Parenting styles (responsive, domineering, and neglectful) and family income affect development. Thus, responsive parenting has a positive impact on children’s life skills. Parental environments and siblings are also valuable in bridging educational gaps, but limited knowledge of available resources and inconsistent finances pose substantial challenges, especially for unemployed single parents without support (25). Factors such as caregiving principles, child safety, and trust in professionals influence parents’ decisions (24).
4. Socioeconomic and Cultural Influences: Mothers of children with SLD report increased levels of stress. Hence, Chinese parents feel more stressed compared to Malay and Indian parents. Higher SES is correlated with increased stress (29). Whereas basic stability is a challenge for certain populations. Along with the lack of knowledge available for children with SLD, financial instability is a significant obstacle (30). This implies that the cause of stress varies according to SES, with survival anxiety in lower SES groups and performance anxiety in higher SES groups.
5. Balance between work and life: For parents of children with LD, employment serves as a financial support and also a source of emotional well-being and stress reduction. According to Freedman et al. (26), working parents need a sense of control and purpose to be appreciated and make a difference at work with expectations comparable to those without these challenges. Al-Yagon and Cinamon (12) indicated that mothers of SLD children experience a higher level of family–work conflict, though they typically encounter lower levels of work–family conflict.

Similarly, Towers (31) examined the paternal perspective, reporting that 60% of fathers are motivated to spend time with their child, but many face acceptance issues, lack of close companionship, and health impacts. Due to this, fathers tend to make significant work changes due to their child’s SLD, and many are unaware of their rights to unpaid leave.

## Discussion

The aim of the review is to synthesize the current literature on the challenges faced by parents as they navigate their professional commitments and the care demands of children with LD. Following primary and secondary screening in accordance with PRISMA guidelines, a total of 15 studies were selected for inclusion in the systematic review. The discussion is structured around five primary themes:

1. Parenting difficulties and Stressors: The evidence of the study posits that caregivers experience positive and negative unintended outcomes, including parental guilt, extreme stress, and marital distress. This pressure affects family dynamics in unexpected ways, leading to the emergence of various coping strategies (22). This view was supported by the study by Kandel and Merrick (32), which depicts that both parents go through significant stress as they get used to providing care. These difficulties intensify stress by balancing work and caregiving responsibilities, which also has an impact on mental health and disruption of the family environment (33). A distinct variation emerges in a study conducted by Karande et al. (34) that indicates mothers of children with SLD had particular, clinically significant anxiety levels that are closely linked to their child’s academic performance. Tkalcic et al. (35) corroborate these perspectives, depicting that parents of children with disabilities endure a “double burden,” wherein the demands of caregiving significantly undermine their psychological well-being, often exceeding the stress levels experienced by parents of typically developing children.
2. Coping strategies and Resilience: Despite stress being constant, the review reveals significant variation in the usage of different coping mechanisms by the parents. Anuar et al. (20) noted that parents heavily rely on emotion-based coping strategies. This illuminates the intricacies of parents’ coping techniques for managing stress. In addition, Chang and Hsu (27) discovered that parents who feel under pressure to seek social assistance often display negative feelings, including anger and worry. They prefer informal support systems, such as friends, family, and colleagues, to professional experts. Heiman (23) states that, despite these difficulties, many parents maintain regular routines and place a strong emphasis on positivity, acceptance, and faith in their child’s future.
3. Parenting Styles and Developmental Milestones: The link between parenting style and child outcomes was demonstrated to be complex and unclear. According to Paul and Babu (28), children with SLD benefit from responsive parenting in terms of their development of life skills. This suggests that parenting style is not the only factor influencing success. However, Taderera and Hall (25) stated that environmental constraints, such as limited information and uncertain funding, make it difficult to provide this assistance.
  This complexity is supported by external literature, such as that reported by Tkalcic et al. (35), who found that the family environment is strongly influenced by external factors, including parents’ occupational status and social support systems. Regardless of the child’s particular deficiencies, their data indicate that parents are better able to deliver positive parenting when they receive external support.
4. Socioeconomic and Cultural Influences: Parents from higher SES backgrounds are more likely to feel stress than parents from middle or lower SES backgrounds, while Chinese parents reported higher levels of stress than Malay and Indian parents (29). Whereas Sreedevi (30) reported that families from lower socioeconomic backgrounds have a major challenge due to financial uncertainty. These findings are consistent with those by Karande et al. (34), who found that the “invisible” aspect of SLD indicates significant uncertainty about the child’s future social position, particularly among educated, upper-middle-class mothers. Thus, it indicates that stigma is a source of stress that affects people from all socioeconomic backgrounds since cultural norms typically limit pecuniary benefits.
5. Balance between work and life: Al-Yagon and Cinamon (12) indicated that mothers of children with SLD experience greater family-to-work conflict due to caregiving responsibilities. In contrast, mothers of children without SLD often experience less work–family conflict. This aligns with resource scarcity theory, which asserts that SLDs’ “invisible” cognitive demands deplete the resources necessary for work performance, whereas employment serves as a major source of purpose in life, autonomy, and emotional well-being for parents (26). This view was supported by the recent study by Tkalcic et al. (35), which examined the association between well-being and employment. The data indicate that working parents encounter difficulties in navigating logistical obstacles, yet report increased life satisfaction as a source of social integration. This demonstrates that although work protects against isolation, it necessitates an ongoing, frequently stressful balance between caregiving and professional responsibilities.
  This review emphasizes the frequently disregarded experiences of parents raising children with SLD by drawing on data from a variety of studies. These parents deal with a variety of difficulties brought on by mental stress, different coping mechanisms, and social expectations. Cultural settings and parenting styles further influence their experiences. The findings highlight the value of specialized support networks that address the interconnections of parents’ personal and professional lives.

### Limitations

This systematic review has the following limitations. First, research examining various developmental disabilities (such as autism or intellectual disabilities) was carefully excluded unless disaggregated data were provided to guarantee specificity to SLD. Thus, because the inclusion criteria emphasized SLD, the literature was significantly reduced to a final sample of 15 studies. As a result, the findings may have narrower generalizability than reviews that focused on a broader range of developmental disabilities.

Second, it lacks evidence of a causal association between caregiving demands and professional commitments, as the majority of the included studies employed cross-sectional designs. To precisely map how these career-care conflicts change over the course of a family’s existence, longitudinal research is necessary. Third, we acknowledge that excluding gray literature, such as conference proceedings, dissertations, and unpublished reports, was a limitation; however, it was intended to ensure the inclusion of high-quality, peer-reviewed evidence.

In addition, only English-language studies were included in the review, which may have excluded pertinent information from non-English-speaking countries. Moreover, the absence of a pre-registered protocol [International Prospective Register of Systematic Reviews (PROSPERO)] is acknowledged as a limitation to the transparency of the review process.

### Implications and future direction

This review helps employers, healthcare professionals, and lawmakers understand the interplay between job and parental duties. This will also play a role in shaping the parental-mediated intervention, supportive legislation, and targeted support networks to alleviate career pressures and foster a healthier work–caregiving balance.

The impact of managing work–life demands on employee performance and well-being underscores the importance of vocational and family psychology. Although this review has laid the foundation for understanding the association between parents’ dual roles, future studies must prioritize the development of parental-mediated interventions. Such intervention not only benefits parents by enabling them to serve as a central figure in their child’s development but also equips them with the evidence-based practices to support the child’s growth.

## Conclusion

Parents experience extreme physical and psychological strain while navigating the pressure within their professional commitments and care demands. Families employ a variety of coping mechanisms and rely on both formal and informal support networks (15). Integrating cultural and social factors is critical to developing effective interventions. The next essential shift toward longitudinal studies should prioritize understanding the long-term financial effects of these caregiving experiences (11). The results suggest the need to develop parental-mediated interventions to promote evidence-based practices and supportive environments that help parents effectively reconcile their dual roles.
